# 154. Impact of Rapid Blood Culture Identification (BCID) PCR Panel on Optimal Antibiotic Use in Methicillin-Susceptible *Staphylococcus aureus* (MSSA) Bacteremia

**DOI:** 10.1093/ofid/ofad500.227

**Published:** 2023-11-27

**Authors:** Jahnavi Yetukuri, Dimple Patel, Aiman Bandali, Pamela Giordano

**Affiliations:** Atlantic Health System, Durham, North Carolina; Atlantic Health System, Durham, North Carolina; Atlantic Health System, Durham, North Carolina; Atlantic Health System, Durham, North Carolina

## Abstract

**Background:**

Optimal anti-MSSA antibiotic choices are nafcillin, oxacillin, and cefazolin. Vancomycin, often used empirically for *S. aureus* infections, and other broad-spectrum β-lactam antibiotics, such as piperacillin/tazobactam and ceftriaxone, are linked with suboptimal outcomes compared to anti-MSSA β-lactam therapy. BCID PCR testing allows for faster pathogen identification, which may improve antibiotic use and clinical outcomes. The objective of this study was to assess the impact of BioFire BCID PCR panel implementation on antibiotic use and clinical outcomes in patients with MSSA bacteremia.

**Methods:**

This was a retrospective chart review of adult inpatients with MSSA bacteremia during the pre-PCR (Jun 2018 - Dec 2019) and post-PCR (Jun 2020 - Dec 2021) study periods. Patients were excluded if they did not have PCR testing performed (post-PCR group only), never achieved optimal antibiotic therapy, had a polymicrobial infection, had positive blood cultures in the prior 90 days, underwent penicillin-skin testing or β-lactam desensitization, or were transitioned to comfort care or deceased within 24 hours of the gram stain. The primary endpoint was the difference in time to starting optimal MSSA antibiotic therapy (oxacillin or cefazolin) between the pre-PCR and post-PCR groups. Secondary endpoints included duration of anti-MRSA antibiotic use, in-hospital mortality, hospital and ICU lengths of stay, duration of bacteremia, and 30-day MSSA-related and all-cause readmissions.

**Results:**

A total of 200 patients were included in the study, with 100 patients in each group. Time to optimal therapy was reduced by 19.9 hours in the post-PCR group (p < 0.001). Fewer patients in the post-PCR group were initiated on empiric anti-MRSA antibiotic therapy than in the pre-PCR group (84% vs 98%, respectively, p = 0.001). PCR implementation was also associated with shorter empiric anti-MRSA antibiotic use and duration of bacteremia. There were no significant differences in length of stay, in-hospital mortality, or 30-day MSSA-related or all-cause readmissions (Table 1).

Table 1: Primary and Secondary Endpoints

**Figure:**
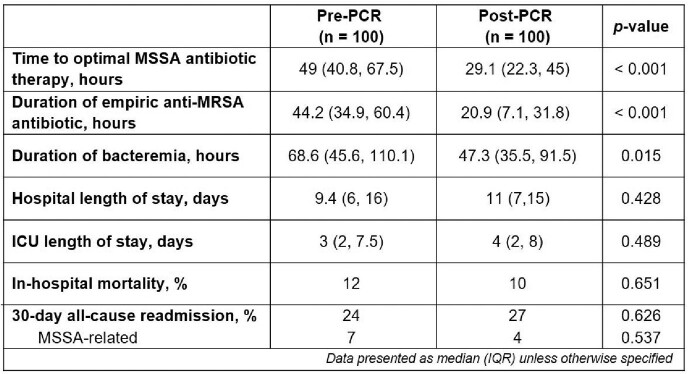

**Conclusion:**

In patients with MSSA bacteremia, BCID PCR panel testing decreased time to optimal MSSA antibiotic therapy and reduced durations of bacteremia and empiric anti-MRSA antibiotic therapy, including vancomycin.

**Disclosures:**

**All Authors**: No reported disclosures

